# Dynamics of Insect–Microbiome Interaction Influence Host and Microbial Symbiont

**DOI:** 10.3389/fmicb.2020.01357

**Published:** 2020-06-26

**Authors:** Ayushi Gupta, Suresh Nair

**Affiliations:** Plant-Insect Interaction Group, International Centre for Genetic Engineering and Biotechnology, New Delhi, India

**Keywords:** plant–insect interaction, endosymbionts, bacterial genome size reduction, insect–microbiome co-evolution, microbial symbiosis, mutualists, plant–microbiome interaction, insect gut microflora

## Abstract

Insects share an intimate relationship with their gut microflora and this symbiotic association has developed into an essential evolutionary outcome intended for their survival through extreme environmental conditions. While it has been clearly established that insects, with very few exceptions, associate with several microbes during their life cycle, information regarding several aspects of these associations is yet to be fully unraveled. Acquisition of bacteria by insects marks the onset of microbial symbiosis, which is followed by the adaptation of these bacterial species to the gut environment for prolonged sustenance and successful transmission across generations. Although several insect–microbiome associations have been reported and each with their distinctive features, diversifications and specializations, it is still unclear as to what led to these diversifications. Recent studies have indicated the involvement of various evolutionary processes operating within an insect body that govern the transition of a free-living microbe to an obligate or facultative symbiont and eventually leading to the establishment and diversification of these symbiotic relationships. Data from various studies, summarized in this review, indicate that the symbiotic partners, i.e., the bacteria and the insect undergo several genetic, biochemical and physiological changes that have profound influence on their life cycle and biology. An interesting outcome of the insect-microbe interaction is the compliance of the microbial partner to its eventual genome reduction. Endosymbionts possess a smaller genome as compared to their free-living forms, and thus raising the question what is leading to reductive evolution in the microbial partner. This review attempts to highlight the fate of microbes within an insect body and its implications for both the bacteria and its insect host. While discussion on each specific association would be too voluminous and outside the scope of this review, we present an overview of some recent studies that contribute to a better understanding of the evolutionary trajectory and dynamics of the insect-microbe association and speculate that, in the future, a better understanding of the nature of this interaction could pave the path to a sustainable and environmentally safe way for controlling economically important pests of crop plants.

## Introduction

Insects represent one of the most diverse and ancient forms of life on Earth and can cause severe devastation if their population size exceeds a particular threshold. On the geological time scale, their existence dates back to the Paleozoic era when Orthopterans and Hemipterans first appeared on Earth (Misof et al., 2014). Since then, they have advanced and have successfully survived even various extreme climatic conditions. Though insects are both ecologically and economically important, in contrast, insect pests, largely a likely creation of man-manipulated habitats, and many of them an outcome of climate change, are involved in the destruction of crops to the extent of more than 20% annually (Deutsch et al., 2018). Further, changing climatic conditions are influencing the migration pattern of insects, timing of their life cycle and their population dynamics. While overcoming these challenges it has also enabled them to expand their host range, affected their behavior and biology, and thereby helping them invade and colonize different agro-climatic zones of the world (Shrestha, 2019). Their large population size combined with short reproductive cycles and high reproductive rates have enabled them to successfully combat all adverse conditions. Owing to their small body weight, light enough to be carried away by the wind currents, they have invaded various parts of the planet and currently they inhabit almost all the ecosystems on Earth.

Besides, the intricate relationship they share with the beneficial microbes has played a crucial part in their diversification and evolutionary success (Janson et al., 2008). Insects are known to be associated with microbes such as bacteria and fungi throughout their evolutionary history. Some bacterial species reside in specialized cells, within the insects, known as bacteriocytes and are referred to as ‘endosymbionts,’ whereas there are others, which are located on the body surface and are called ‘ectosymbionts’ (Thompson and Simpson, 2009). However, they are predominantly present in the digestive tract where they act as key modulators of the diverse lifestyles (both in terms of diet and ecological niches) of their insect host. The gut-microflora of an insect are known to (1) facilitate its feeding even on recalcitrant food; (2) provide immunity and protection against various predators, pathogens and parasites; (3) compensate the nutrient-poor diet (e.g., in the case of sap-sucking insects); (4) mediate inter- and intra-specific communication; (5) control mating and reproductive success; (6) aid digestion and, (7) supply essential amino acids, metabolic compounds and nutrients (Russell et al., 2014; Douglas, 2015; Arbuthnott et al., 2016; Wielkopolan and Obrêpalska-Stêplowska, 2016; Engl and Kaltenpoth, 2018). In fact, Jing et al. (2020) have shown that essential nutrient provisioning is the primary task of symbionts followed by digestion and detoxification. Thus, it implies that insects are highly dependent on their gut microbiome for survival and normal transactions related to their life cycle. Furthermore, based on the degree of dependence, their association can be classified as obligate (or primary) and facultative (or secondary) (Baumann, 2005; Moran et al., 2008). However, there is no clear demarcation between these two categories as facultative bacteria can become obligate under special circumstances (Ferrari and Vavre, 2011).

Together, the endosymbionts and its insect host have formed a very intricate and intriguing relationship, various aspects of which are yet to be explored and understood. Some researchers consider these symbionts as ‘intracellular parasites’ that have hijacked the insect body and thereafter evolved various mechanisms to ensure their survival while providing benefits to their host. However, it is equally probable that the insect initiated this relationship with its microbiome for its survival. Whichever the case, they have now adapted themselves to each other. Bacterial species present within an insect gut can exhibit mutualism, commensalism or could even be pathogenic (Dillon and Dillon, 2004).

Usually, insects initiate an immune response against the pathogenic bacteria but can selectively maintain the beneficial microbes (Mikonranta et al., 2014). Specific functions of symbiotic anti-microbial peptides (AMPs) have been studied experimentally, revealing that they regulate symbiotic interactions by limiting the reproduction of symbiotic bacteria, sometimes transforming them into a differentiated form, and eliminating undesirable, sensitive bacteria (Mergaert, 2018). Several structural families of AMPs are reported from insects that include apidaecin, hymenoptaecin, defensins, cecropins, drosocins, attacins, diptericins, ponericins, metchnikowins, and melittin (Kwong et al., 2017; Wu et al., 2018). In bees, the expression of the AMPs, apidaecin and hymenoptaecin, is up-regulated in gut tissue, upon microbial acquisition (Kwong et al., 2017). However, it is shown that the endosymbiotic bacteria do not induce the antibacterial responses, such as expression of genes coding for AMPs, in insects (Eleftherianos et al., 2013). For instance, *Wolbachia* when present as a facultative symbiont in *Aedes albopictus* does not trigger the synthesis of AMPs (Bourtzis et al., 2000); however, it induces an immune response in *Anopheles gambiae*, which is not its natural host (Hughes et al., 2011). Likewise, the presence of *Serratia symbiotica* in aphids does not alter the expression of defense-related genes (Burke and Moran, 2011). Additionally, it is observed that different symbionts interact differently with the insect immune system; while some can successfully bypass the insect’s cellular immune response others affect the melanization response (a defense mechanism present in insects) (Thomas et al., 2011). Similarly, in this context, recent data seem to indicate several means by which the symbionts are capable of evading insect immunity. However, the processes by which endosymbionts acquired this ability to circumvent the insect immune system or how the insect is able to differentiate between the ‘beneficial symbionts’ and potentially pathogenic ones is still unclear. But what is clear is that upon initiation of symbiotic association, both the participating partners (the bacteria and the insect) undergo several changes, mediated by the action of various evolutionary forces, which possibly endow the bacterial symbionts with the capability to bypass or evade the insect immune system.

Microbial symbiosis involves acquisition, colonization and transmission. While insects readily acquire several bacteria during their life cycle, others are vertically transmitted or inherited. Post acquisition, successful colonization is crucial for their survival and persistence, which, in turn, is highly influenced by the physical and physiological conditions of the insect gut. Thereafter, and especially for obligate endosymbionts that are completely dependent on their host for survival, transmission across generations becomes critical. Therefore, to ensure transmission, microbes have evolved several fascinating mechanisms that will be discussed elsewhere in this communication.

Previous studies have unraveled mechanisms involved in the acquisition, maintenance and transmission of endosymbionts. However, we have limited information regarding the mechanisms that drive this entire transition, i.e., the transition of a free-living microbe to an obligate symbiont residing within an insect. Interestingly, the bacterial genome is known to undergo changes to acclimatize itself to the gut environment. It not only modifies itself but some microbes are even capable of manipulating its insect host for their survival (Yuval, 2017). Because of the increased focus on microbiome in general and microbiome of insects in particular in the past decade, our understanding of insect-microbe interactions has also increased. Researchers have now demonstrated that both the insects and their symbionts are tightly inter-connected at almost every level of their evolution. The insect host is known to play a major role in shaping its microbiome (Engel and Moran, 2013), and these endosymbiotic bacteria have now become such an integral part of the insect’s body, that they co-evolve with their host. This review is an attempt to summarize our current understanding of the fate of microbes inside an insect’s gut, and highlight physical, physiological and functional implications on their insect host. We believe that dissecting the mechanisms directing co-evolution of insect–microbial symbiosis would not only provide us with a better understanding of this association, but information thus obtained could further be applied toward devising sustainable pest management strategies.

## Diverse Forms of Insect–Microbe Associations – From Initiation to Their Diversification

Acquisition of microbes by the insect from the environment is usually the foundation of insect–microbe symbiosis and subsequently, after acquisition, these bacteria undergo a gradual transition from free-living organisms to being intracellular parasites. Once inside the insect’s body, their persistence depends largely on the host’s life cycle. Upon acquiring bacterial species not only do insects change their feeding habits but they also create specialized niches and gut compartments for housing these microbes that enable and promote microbial persistence (Engel and Moran, 2013). Microbial colonization is heavily affected by the physiochemical conditions of the gut, particularly its pH. Insects housing huge microbial communities provide a favorable environment to their bacterial symbionts by providing them with the optimal pH within the gut (Engel and Moran, 2013). Varied types of bacterial endosymbionts present within the insect body have been reported and each with their distinctive features (Table 1).

**TABLE 1:** Types of microbial symbionts of insects and their attributes.

Features	Types of microbial symbionts			References
	Obligate	Facultative	Phytopathogenic	
Acquisition	^a^Maternal	^a^Environmental	^d^Plants via feeding	^a^Baumann, 2005
Localization	^a^Bacteriocytes	^b^Ubiquitously (Hemocoel)/confined (Bacteriocytes)	^c^Salivary glands	^c^Kwon et al., 1999; ^b^Marubayashi et al., 2014
Transmission	^a^Vertical	^a^Horizontal	^d^Horizontal	^d^Chrostek et al., 2017
Key functions	^a^Nutrition provisioning	^e^Digestion and detoxification	^d^Enhances virulence, facilitate feeding	^e^Moran et al., 2005
Genome size	Highly reduced	Normal	Normal	Nakabachi et al., 2013
Host dependency	Mutualists	Mutualists, Commensals or pathogenic	Mutualists, Commensals or pathogenic	Moran et al., 2008

*The reference citations and references are indicated with superscript letters when the provided information is derived from more than one reference.*

The obligate mutualists upon entering the insect’s body, localize themselves inside bacteriocytes, provide benefits and fitness advantage to the host, and are transmitted maternally across generations. They establish a very stable mutualistic association with their host. They supplement the nutritional requirement of their host by synthesizing essential amino acids and rare vitamins especially for the hemimetabolous sap-sucking insects that feed on nutrient-poor diets. For instance, in *Buchnera aphidicola*, an obligate symbiont of aphids, and probably the most studied model, it has been shown that the bacteria fulfills the nutritional requirement of the insect host to an extent that its removal dramatically affects aphid survival and fecundity (Feng et al., 2019). Similarly, symbiosis between the sap-feeding insect *Megacopta cribraria* and its primary bacterial symbiont, *Candidatus* Ishikawaella capsulate, is essential for host survival to adulthood (Couret et al., 2019). And it is just not the insect that is dependent upon these obligate symbionts but also many long-term obligate symbionts over time have become highly dependent on their insect host. For example, *Buchnera* that lives in a metabolic collaboration within the pea aphid (*Acyrthosiphon pisum)* has lost genes for the synthesis of various branched-chain amino acids (such as isoleucine, valine, and leucine). *Buchnera* is, therefore, entirely reliant on its insect host for the supply of these amino acids, which are crucial in vitamin biosynthesis pathways (Wilson et al., 2010; Hansen and Moran, 2011; Russell et al., 2013).

In contrast, the facultative microbes exhibit an entirely different scenario as some of them are vertically transmitted e.g., *Wolbachia, Spiroplasma*, and *Cardinium*, whereas others are acquired afresh after every generation such as *Burkholderia* and *Serratia*. Further, in several cases, e.g., in the Dipteran pest of rice, the Asian rice gall midge, it has been shown that the community structure of several facultative microbes is highly influenced by the host’s developmental stage and diet (Ojha et al., 2017). Besides, the facultative symbionts pre-dominantly assist their insect hosts in digestion and xenobiotic detoxification e.g., some species of *Pseudomonas*, a gram-negative Gamma-proteobacteria found in *Spodoptera frugiperda*, are involved in providing pesticide resistance to their hosts (de Almeida et al., 2017); *Serratia grimesii*, in nematodes, possesses genes involved in the degradation of phytotoxins such as terpenes; and *Candidatus* Ishikawaella capsulate is known to metabolize alkaloids in stinkbugs (Itoh et al., 2018). Moreover, several groups of vertically transmitted facultative endosymbionts such as *Wolbachia, Rickettsia, Arsenophonus, Spiroplasma*, and *Cardinium* are involved in sex determination and are known to induce sexual aberrations across various insect orders (Kageyama et al., 2012). Therefore, some facultative microbes are beneficial for the host (at least under certain circumstances) whereas some are commensals and others even pathogenic. They are known to inhabit various parts of an insect’s body and represent the dynamic component of the insect’s microbiome. While some are localized to the hemocoel and are present ubiquitously (scattered pattern), others are restricted to the bacteriocytes (confined pattern) (Marubayashi et al., 2014). Unlike obligate symbionts that mostly exhibit transovarial transmission, the facultative symbionts have evolved various fascinating mechanisms to ensure their transmission and propagation inside an insect’s body. For instance, *Sodalis glossinidius*, a facultative symbiont of the tsetse fly has evolved the capacity to be transmitted through transovarial transmission via haemolymph (Cheng and Aksoy, 1999), or vertically to the intrauterine larvae via milk gland secretions and in some instances, horizontal transmission during mating (De Vooght et al., 2015) was also observed. Apart from this, there are substantial number of interesting studies indicating the nature of various bacterial symbionts across different insect orders, and these are summarized in Table 2.

**TABLE 2:** List* of bacterial species and type of their associations with insects; their known mode of acquisition, localization and transmission.

Bacterial symbiont	Nature of association	Insect host(s)	Mode of acquisition	Localization within the host	Mode of transmission	References
*Buchnera aphidicola*	Obligate Mutualism	Aphids	Inherited	Bacteriocytes	Transovarial	Baumann, 2005
*Carsonella ruddii*	^a^Obligate mutualism	^a^Psyllids	^b^Inherited	^b^Bacteriocytes	^b^Transovarial	^a^Thao et al., 2000; ^b^Thao et al., 2001
*Blochmannia floridanus*	Obligate mutualism	Carpenter ants	Inherited	Somatic cells surrounding ovarioles	Transovarial	Kupper et al., 2016
*Wigglesworthia glossinidia*	Obligate mutualism	Tsetse flies	Inherited	Bacteriocytes	Transovarial	Bing et al., 2017
*Serratia symbiotica*	Facultative commensalism	Aphids	Environmentally acquired	NA	Horizontal transmission	Pons et al., 2019
*Regiella insecticola*	Facultative commensalism	Aphids	Inherited	Bacteriocytes, Haemolymph	Transovarial	Vorburger et al., 2010
*Hamiltonella defensa*	Facultative Commensalism	Aphids, Whiteflies	Acquired and Inherited	Sheath Cells, Secondary Myocetocytes, Haemolymph	Horizontal and Maternal	Marubayashi et al., 2014
*Portiera aleyrodidarum*	Obligate mutualism	Whiteflies	Inherited	Bacteriocytes	Transovarial	Santos-Garcia et al., 2015
*Tremblaya princeps*	Obligate mutualism	Mealy bugs	Inherited	Bacteriome	Transovarial	López-Madrigal et al., 2013
*Sodalis glossinidius*	Secondary facultative	Tsetse flies	Inherited and Acquired	Numerous tissues	^a^Milk gland, ^b^Transovarial, and ^a^Mating	^a^De Vooght et al., 2015 ^b^Cheng and Aksoy, 1999
*Baumannia cicadellinicola*	Obligate mutualism	Sharpshooters	Inherited	Bacteriocytes	Transovarial	Wu et al., 2006
*Sulcia muelleri*	Obligate mutualism	Sharpshooters	Inherited	Bacteriocytes	Transovarial	Moran et al., 2005
*Nardonella* sp.	Obligate mutualism	Weevils, Beetles	Inherited	Bacteriocytes	Transovarial	Kuriwada et al., 2010
*Candidatus Arsenophonus arthopodicus*	Facultative commensalism	Louse flies	Inherited	Intestine wall (bacteriocytes), Lumen of milk glands	Transovarial	Nováková et al., 2015
*Wolbachia* sp.	Facultative parasite	Various insects	Inherited	Bacteriocytes, extracellularly scattered	Transovarial	Miller, 2013
*Rickettsia* sp.	Facultative parasite	Various insects	^b^Inherited	Extracellularly Scattered, Bacteriocytes	Transovarial	^a^Behar et al., 2010 ^b^Gottlieb et al., 2006
*Spiroplasma* sp.	^a^Facultative parasite	Various insects	Inherited	Haemolymph, Endocellularly localized	Transovarial	Bové, 1997
*Cardinium* sp.	Facultative parasite	Planthoppers	Inherited	Gut, testicles, oocytes, glands	Transovarial	Gonella et al., 2011
*Ishikawaella capsulata*	Obligate mutualism	Plataspid stinkbugs	Inherited	Extracellular (midgut)	Capsule	Nikoh et al., 2011
*Rosenkranzia clausaccus*	Obligate mutualism	Stinkbugs	Inherited	Midgut crypts	Egg smearing	Hayashi et al., 2015
*Rhodococcus rhodnii*	Facultative mutualism	Assassin bugs	Environmentally acquired	NA	Coplophagy	Kikuchi, 2009
*Serratia marcescens*	Pathogenic	Grassland locusts	Soil	Fat bodies	Insecticidal properties	Tao et al., 2006
*Burkholderia* sp.	NA	Bean bugs, stinkbugs	Environmentally acquired	Crypts at posterior midgut region	Horizontal transmission	Kikuchi and Yumoto, 2013
*^a^Rickettsia* sp., *^b^Cardinium* sp., *^a^Wolbachia* sp.	Facultative parasites	Leafhoppers	^a^Inherited, acquired	^a^Intracellular and Scattered	Transmitted to plants	Nakamura et al., 2009; Gonella et al., 2015
*Candidatus liberibacter psyllaurous*	Facultative	Tomato psyllids	Acquired during feeding	Extracellular	Vector	Hansen et al., 2008
*Moranella endobia*	Obligate mutualism	^b^Mealy bugs	^b^Inherited	^a^Inside Tremblaya *princeps* cells	Maternal	^a^López-Madrigal et al., 2013 ^b^Husnik and McCutcheon, 2016
*Blochmennia floridanus*	Obligate commensalism	Carpenter ants	Inherited	Oocytes, Enterocytes of midgut tissue (Bacteriocytes)	Maternal	Zientz et al., 2006
*Xenorhabdus nematophilus*	Pathogenic	Wax moths	Acquired	Haemolymph	Toxic	Mahar et al., 2005
*Photorhabdus luminescens*	Pathogenic	Tobacco hornworm	Acquired	Haemocoel	Toxic	Münch et al., 2008
*Serratia marcescens*	Opportunistic pathogen	Fruit flies	Acquired	Gut, body cavity, intestinal epithelial cells	Toxic	Nehme et al., 2007
*Serratia entomophila*	Pathogenic	Grass grubs	Feeding	Digestive tract	Toxic	Hurst et al., 2004
*Enterobacter aerogenes, Bacillus cereus, Bacillus sphaericus, Serratia* sp., *Klebsiella* sp., *Morganella* sp.	Temporal association	Neuropterans (Antlions)	NA	NA	Toxic	Nishiwaki et al., 2007
*Erwinia chrysanthemi*	Specific pathogen	Aphids	Septic injury, oral injection	NA	Toxic	Costechareyre et al., 2012

*Though most of the bacterial species can be found to be associated with more than one insect species, the predominant host is listed here. NA, data not available. The reference citations and references are indicated with superscript letters when the provided information is derived from more than one reference.

As a result of the feeding process, besides obligate and facultative symbionts, several phytopathogenic microbes are also found in insect bodies. However, plants, being immobile, become a major obstacle in the transmission of these phytopathogenic bacteria. This led to the dependency of these microbes on vectors, which are usually insects, for their dispersal and propagation. And consequently, initiating an association of these microbes with their insect vector and in turn shaping this complicated relationship that is currently observed between them. These bacteria not only actively interact with their insect host but also modify it for their own benefit. Some microbes can multiply within its insect vector (propagative) while some cannot (non-propagative). This implies that besides serving as the vector, the insect also serves as an alternate host for these bacteria (Nadarasah and Stavrinides, 2011). Some microbes once acquired by the vector are readily transmitted to the host plant (semi-persistent, non-circulative) whereas others circulate through the body of the insect and are transmitted only after a latent period (persistent, circulative transmission) (Perilla-Henao and Casteel, 2016). Upon entering the insect’s body they migrate to the midgut or hindgut epithelium and are subsequently released into the haemolymph. From the haemolymph, they enter the salivary gland and are transmitted to the plant during the feeding process (Kwon et al., 1999). In turn, insects have also evolved mechanisms that enable them to tackle these pathogens and derive certain advantages out of this insect-microbe interaction. Though the mechanisms behind the co-evolution of these microbes and their insect vector are both fascinating and crucial for the understanding of microbes–insect–plant interactions, it will not be discussed further as it is beyond the scope of this review.

As discussed in earlier sections and evident from the data summarized in Table 2, these endosymbionts have, over time, evolved various mechanisms that are critical for sustenance within an insect body. The occurrence of these varied types of associations raises the question, what led to this diversification? Why and how did some bacteria become an obligate intracellular symbiont in some insects while it remained facultative in others? What determines the nature of association of any microbe for a particular insect? It could be the likely outcome of its (bacterial) functional capacities (the capacity to fulfill the host’s requirement) and capabilities (an important aspect being the capability to evade insect’s immune system). Furthermore, it could also be determined by the insect host based on the extent of its dependency on that bacterium. However, the possibility that these associations are made under selective influence, where an insect found in a particularly harsh environment is forced to form an association with the microbe to overcome the immediate biotic and abiotic stresses, cannot be negated. Despite several studies, we still have very limited information regarding mechanisms that led to the evolution and eventually diversification of these associations.

## Fate of Microbes Within an Insect Body – Their Journey of Transition From Free-Living to an Obligate Symbiont

The fitness of an organism and its success at any given point of time depends upon its genome flexibility that provides it with the capability to adapt and adjust as and when required depending on the environment. However, it is ironic to note that while organisms strive toward achieving genome stability, this drive could also become a reason for its demise or extinction. Although genome stability allows maintenance of adapted phenotypes, it is also a major obstacle in the evolution of novel and superior traits that enable an organism to tolerate change (Schubert and Vu, 2016). Evolutionary data suggest that several species have become extinct because of their inability to cope well with the changing environmental conditions. However, insects, due to their genome flexibility, can rapidly adapt by undergoing modifications in their genome size, composition and its architecture (Robertson, 2005) and thereby helping it overcome/survive the adverse conditions. Moreover, owing to the large population size, any beneficial variation, induced by the evolutionary forces, gets fixed in an insect population rapidly.

**FIGURE 1:**
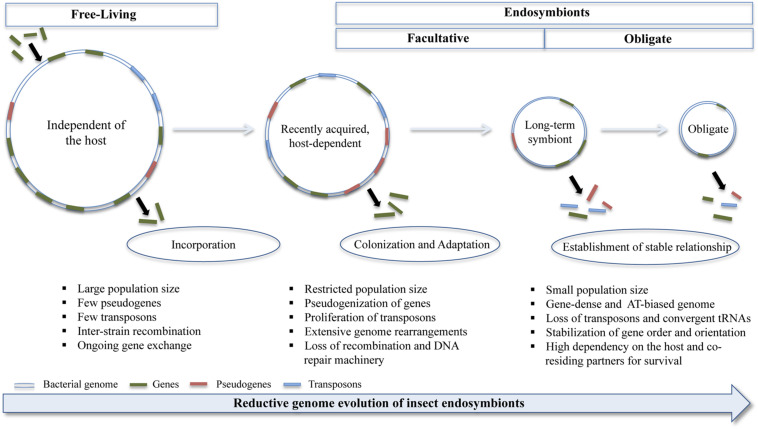
Diagrammatic representation of steps involved in the transition of free-living bacteria to obligate insect endosymbionts. The host-restricted bacteria undergo changes in its genomic size, composition and architecture; and these are brought about by excessive gene loss, chromosomal rearrangements, proliferation of transposons and pseudogenization of several non-essential genes during the adaptation stage and these processes are attenuated on transformation to obligate symbionts (see text for details). Objects in the figure are not drawn to scale.

In addition, it harbors these endosymbiotic bacteria that it has remodeled for its benefit. It has been shown that the several bacterial species that are present within an insect body differ remarkably from their free-living counterparts (Kikuchi, 2009). Studies have revealed that the genomes of endosymbiotic bacteria carry signatures not only signifying its phylogenetic position, but also revealing the kind of lifestyle to which it has adapted. Various genome-specific signatures such as base composition, GC-skew, purine-pyrimidine ratio, dinucleotide abundance, codon bias, oligonucleotide composition etc. have been identified from the endosymbiotic bacterial genomes (Dutta and Paul, 2012). Besides, the bacterial species present in an insect gut have a highly reduced genome (i.e., they have small, gene-dense genome) as a result of sequential gene loss (Figure 1; Wernegreen, 2002). It has been demonstrated experimentally by several research groups that endosymbiosis involves massive genomic rearrangements brought about primarily by mobile element proliferation and pseudogenization of non-essential genes (Van Ham et al., 2003; Pérez-Brocal et al., 2006; Moran et al., 2009). In aphids, it is shown that the recently incorporated *Serratia symbiotica* (genome size ∼2.79 Mb) is at the pseudogene proliferation stage with ∼550 pseudogenes as opposed to ∼12 pseudogenes found in its free-living relative, *S. proteamaculans*; whereas the other co-residing ancient endosymbiont, *Buchnera aphidicola* (genome size ∼0.652 Mb) has undergone pseudogenization of several non-essential genes (Nicks and Rahn-Lee, 2017). However, an exception to this is *Sodalis glossinidius*, a facultative bacterial symbiont of tsetse flies, whose genome analysis revealed large-scale and significant expression of pseudogenes and thereby suggesting that it is a recent acquisition by these insects (Goodhead et al., 2020). Furthermore, *Carsonella*, an obligate symbiont of psyllids, and one of the smallest known symbionts in terms of its genome size (i.e., ∼173 kb) has undergone extensive gene loss making it entirely reliant on its host for survival (Thao et al., 2000). These findings have been further corroborated by correlation analysis, carried out by Fisher et al. (2017) on 58 obligate bacterial symbionts found in 89 host species including plants, fungi, insects, and other arthropods, that suggested a negative correlation between host dependence and symbiont genome size. Thus indicating that genome reduction due to gene losses lead to complementation and functional redundancy, which reinforces the inter-dependency of microbes on one another and their host. And this is one of the widely accepted phenomena that are known to occur within an insect gut.

**FIGURE 2:**
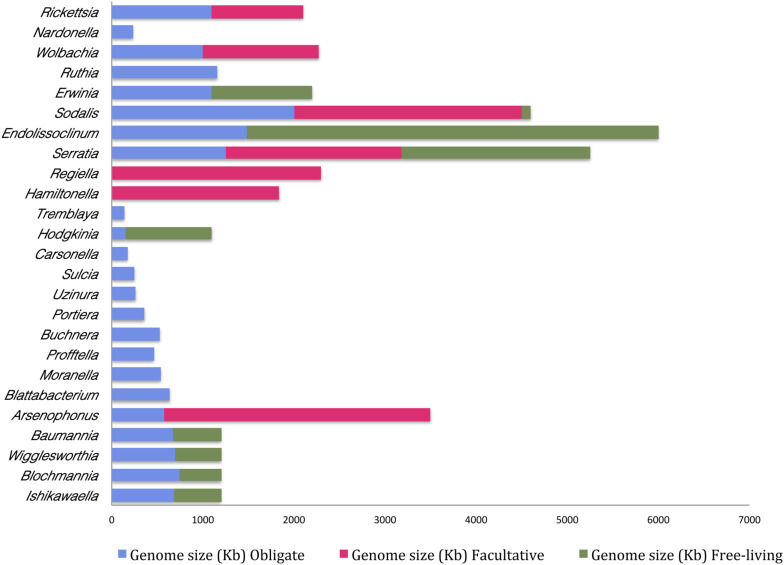
Differences in genome sizes of the free-living bacteria and their cognates in a facultative or obligate symbiotic relationship with an insect host. Names on the *Y*-axis are the bacterial species and genome sizes (Kb) are indicated on the *X*-axis. (This figure is based on published information and the relevant references are provided in Supplementary Table T1).

Moreover, it appears that the extent of genome reduction depends upon the nature of association. It is generally observed that the bacteria under obligate symbiotic association have a comparatively smaller genome than when it occurs as a facultative symbiont. For instance, *Arsenophonus* sp. when found as an obligate symbiont in *Riesia pediculicola*, has a genome of ∼570 kb while the one that is associated with *Nasonia* spp. (as a facultative symbiont) has a genome size of approximately 3500 kb (Nováková et al., 2016). This suggests that the smaller the genome size higher is its dependency on the host. And, it also indicates that gene loss is probably one of the primary reasons for the transition of any facultative symbiont to an obligate symbiont inside the host. This phenomenon is comparable to evolution of mitochondrial and chloroplast DNA within a eukaryotic cell, which represents a classical case of genome reduction during symbiosis. Mitochondria and chloroplasts represent the ultimate outcome of ‘reductive evolution’ as they have undergone up to 95% reduction in their genome upon transition from free-living to obligatory intracellular parasite (Gray, 2012). Obligate and facultative symbionts possess one of the smallest genomes when compared to their free-living forms (Figure 2 and Supplementary Table T1).

Besides, these studies also indicate that the symbionts of the recently formed associations with hosts are slightly reduced in their genome size whereas the ones that represent an older association have a highly reduced genome. For instance, *Serratia* that is known to be associated with *A. pisum* for over 100 million years has one of the highly eroded genomes ranging from 0.65–1.76 Mb as opposed to 5.11–5.45 Mb genome of its free-living counterpart (Richards et al., 2010). Likewise, *Buchnera aphidicola*–aphid symbiosis originated about 200 million years ago, and over time *Buchnera* genome has been drastically reduced to less than 0.7 Mb inside its insect host (Gil et al., 2002). Furthermore and in congruence, the last decade has witnessed several studies in this regard (Supplementary Table T1) and results from such studies point toward the fact that the genome size of a bacterial endosymbiont is inversely proportional to the time for which it has been associated with the host.

Researchers have shown that the endosymbiotic bacteria have undergone drastic genetic, phenotypic and biochemical changes as observed when compared with their free-living counterparts (Figure 1). And the gene-loss observed in the bacterial symbiont, while in association with its host, appears to be a non-random and a continuous phenomenon (Sabater-Muñoz et al., 2017). It has been observed that the gradual decrease in the genome size upon transition from free-living to obligate form, is accompanied by several other changes in genome characteristics including decrease in its GC-content and number of coding genes (Van Leuven and McCutcheon, 2012); reduction in the number of copies of *rRNA*, *tRNA* and other non-coding genes (Manzano-Marín and Latorre, 2016); proliferation of mobile elements at first (during facultative association) and their subsequent loss upon transition to an obligate symbiont (Belda et al., 2010). Genome reduction is also known to influence translation in endosymbionts where convergent tRNA loss has been observed in microbes that have undergone severe genome reduction. For instance, loss of modified nucleoside pathways, introduction of high AT-bias that resulted in reduced tRNA thermostability and various alternations in tRNA features crucial for translation, have been reported for *Buchnera* symbiont of aphids (Hansen and Moran, 2012).

In addition, it is often noticed that the bacterial partner usually retains the genes involved in symbiotic functions (McCutcheon, 2016). For example, *Portiera* has retained genes encoding essential amino acids and carotenoids but lacks several vitamin and co-factors producing genes which are compensated by the other symbiont, *Hamiltonella*, that is known to co-occur with *Portiera* within their common insect host, *Bemisia tabaci* (Rao et al., 2015). Together they also fulfill the nutritional requirements of their host. Similarly, to obtain nitrogen from the uric acid stored in the fat bodies, the cockroach (*Blattella germanica*) utilizes urease produced by its primary endosymbiont *Blattabacterium cuenoti* (López-Sánchez et al., 2009; Patiño-Navarrete et al., 2013); *Candidatus* Portiera aleyrodidarum supplements the diet of their phloem-feeding hosts by supplying essential amino acids and vitamins (Zientz et al., 2004; McCutcheon and von Dohlen, 2011); *Sulcia muelleri* and its co-resident *Hodgkinia cicadicola* synthesize essential amino acids in cicadas (McCutcheon et al., 2009). Interestingly *Cardinium*, in spite of its reduced genome size with several genes coding for various metabolites lost, encodes the complete biosynthetic pathways for biotin and lipoate, which are crucial for its host’s nutrition (Zeng et al., 2018). And not only are there examples of host-endosymbiotic metabolic collaboration but the endosymbiotic bacteria are also known to complement each other in several insects. For instance, *Moranella endobia* and *Tremblaya princeps* are known to complement each other within mealybugs (López-Madrigal et al., 2013); *Serratia* complements *Buchnera* in aphids (von Dohlen and Moran, 2000); *Carsonella eucalyptia* (primary symbiont) and *Heteropsylla cubana* (secondary symbiont) exhibits strict complementarity in the biosynthesis of tryptophan in psyllids (Sloan and Moran, 2012); *S. muelleri* supplies amino acids to various co-residing symbionts (Rao et al., 2015).

It is also interesting to note that the bacterial species that complement each other the most, co-occur inside the same bacteriocyte. For instance, *Portiera* and *Hamiltonella* have undergone genome reduction, exhibit metabolic complementation and are mostly present inside a single bacteriocyte in their host, *Bemisia tabaci* (Rao et al., 2015). Similarly, Alonso-Pernas et al. (2017) studied the bacterial communities localized to the hindgut wall in the forest cockchafer, *Melolontha hippocastani*, and have shown that the composition of bacterial community depends on the insect’s life stage. Further, their data revealed the occurrence of specialized bacterial niches (‘pockets’) attached and connected to both sides of the distal part of the hindgut wall. In addition, they have reported that the Poly-β-hydroxybutyrate (PHB) accumulating bacteria *Achromobacter* sp., was co-localized within these ‘pockets’ and, therefore, it was speculated that the presence of this polymer might play a role in the colonization of these specialized niches. These studies indicate the possibility that the microbes within an insect are compartmentalized into separate bacteriocytes based on their functional capabilities and complementation. However, due to the lack of sufficient and reliable experimental evidences, this is merely a conjecture.

Currently, there is enough information available regarding the nutritional and metabolic collaboration among bacterial endosymbionts of insects. However, it is still uncertain how these intricately intertwined metabolic networks have evolved. Is it a random phenomenon driven by evolutionary forces such as mutation and genetic drift or is guided by selection? The last decade has witnessed several studies in this regard that indicate the complexity and intricacy of the evolutionary mechanisms that are responsible for shaping an insect’s microbiome and will be discussed in the following section.

## Mechanisms Driving the Transitional Evolution of Insect Endosymbionts

It is observed that the parasites and symbionts undergo ‘simplification’ rather than evolving complex metabolic pathways inside their insect host and ‘genome reduction’ is considered a dominant mode of evolution of endosymbionts (Wolf and Koonin, 2013). Taken together, studies strongly indicate the likelihood of ‘community-level selection’ being imposed on these bacterial species residing within their insect host. The famous ‘black-queen hypothesis (BQH)’ proposed by Morris et al. (2012) appears to hold true in several cases of insect–microbe symbiosis. According to BQH, insect–microbiome dependencies and collaborations are a consequence of selection-driven reductive genome evolution of endosymbiotic bacterial species. Lee and Marx (2012) have already shown the prevalence of selection-driven genome reduction in experimental populations of *Methylobacterium extorquens*. They observed parallel deletions (resulting in ∼10% reduction in genome size) in a megaplasmid present in this bacterium when cultured for ∼1500 generations under constant selection under laboratory conditions. Their data, therefore, provide the ideal evidence to corroborate the concept of selection-driven reductive evolution of gut endosymbionts.

Moreover, the gene loss in these symbionts confers a selective advantage by conserving energy and resources where gene function is dispensable. The bacterial species within an insect host are functionally synchronized and thereby reducing the pressure on individual bacterial species to maintain its complete metabolic network. This is achieved when the co-residing bacterial species become part of a diversified metabolic network working in partnership with each other while not subjecting their insect hosts to additional metabolic load. Therefore, this ‘adaptive genome streamlining’ of bacterial endosymbionts could prove to be highly beneficial especially for the insect host. In addition, these changes that occur in the bacterial genome, likely endow the bacteria with structural and functional stability. The bacterial genome, upon losing non-coding DNA and genes not critical for symbiont function, becomes highly stable with regard to gene order and orientation (Sabater-Muñoz et al., 2017). This implies that these changes are non-random and adaptive and are, therefore, likely to be driven by selection.

In contrast, this could also be brought about by the action of genetic drift. According to “Muller’s ratchet hypothesis” the endosymbionts evolve under the influence of drift, as they are believed to experience a relaxed selection imposed by small population bottlenecks within an insect gut (Pettersson and Berg, 2007). The effect of drift and bottlenecks is profoundly exaggerated in the symbionts that solely rely on vertical transmission and there is no horizontal transmission for compensation (Mira and Moran, 2002). In addition, the asexual mode of reproduction in symbiotic bacterial species results in their isolation from the recombination processes resulting in rapid genome degradation (Moran and Plague, 2004). Therefore, the degenerative trajectory of the bacterial genomes present inside insects could be very well explained by the reduced efficiency of natural selection. Also, it has been observed that the endosymbionts have genomes that are highly AT-rich. Usually, selection force and recombination events eliminate the AT-rich sequences and favor GC-rich coding gene sequences (Bobay and Ochman, 2017). But the fact that the genomes of endosymbionts are AT-rich, supports the hypothesis that the evolution of endosymbionts is under weak-selection combined with the absence of genetic recombination.

However, it is also worth considering that the early events involving gene-inactivating mutations and replication slippage are biased toward the GC-rich component of the bacterial genome (i.e., mostly in the genic regions) (Clayton et al., 2016). However, once it has adapted to the insect gut, these events could be deleterious. Therefore, possessing an AT-rich genome could also be an adaptive trait, conferring stability to the bacterial genome.

Additionally, another interesting observation is that symbionts often lose DNA replication and repair mechanisms along with the recombination system quite early in their association with the host (Moran et al., 2008). And this gene loss is not random but is both pre-determined and adaptive. Loss of DNA repair pathway leads to increased deleterious mutations, and in turn, gene inactivation. This also increases the scope for introducing variation in the genome especially when the organism is struggling to adapt to a certain lifestyle within an insect host. A study by Giraud et al. (2001) showed that the mutated phenotypes that arise in the natural and laboratory-based experimental populations of bacteria are highly similar to the mutated phenotypes present within an insect gut. This means that these mutations are advantageous as they facilitate symbiont adaption to the insect gut environment. Moreover, it does correlate with the loss of genes that encode products targeted by an insect’s immune system as a part of the adaption of bacteria to the insect’s body (Ratzka et al., 2012). Similarly and in congruence, data obtained by Toft and Fares (2008) while studying the evolution of flagellar assembly pathway in genomes of endosymbiotic bacteria, suggest that flagellar genes in endosymbiotic bacteria, of insects, belonging to Gamma-proteobacteria, have functionally diverged to adapt to the new environment and become specialized in exporting proteins from the bacterium to the host. These processes are too intricately organized to occur randomly and it is hard to comprehend that merely random processes such as mutations and drift are sufficient for driving transition of any free-living bacteria to an obligate endosymbiont. However, owing to limited information, it is still uncertain whether the reductive evolution of endosymbionts is driven by genetic drift or guided by selection or is a consequence of combination of both the evolutionary forces acting upon it.

Furthermore, although it seems reasonable to believe that the bacterial community within the insect gut experience relaxed selection but what about the selection pressures experienced by the host? Importantly, it is worth noting that changes occurring in the bacterial genome mostly benefits the host. Moreover, now there are indications that various environmental constraints, dietary shifts, change in ecological niches due to colonization and invasion of new habitats, could dramatically affect the bacterial community within the insect (Ng et al., 2018). In fact, insects adapt to various environmental fluctuations by modifying their microbiome. For instance, *Drosophila melanogaster* survivability under extreme conditions is determined by its microbiome composition. Moghadam et al. (2018) showed that reshaping the gut microbiota of *D. melanogaster* immensely affected its thermotolerance capacity. Therefore, the role of insect host in shaping its microbiome cannot be negated. The insect and its bacterial symbiont are so tightly coupled that the selection pressures experienced by the insect host could also play a major role in channeling the direction of evolution of its endosymbiotic bacteria. Rennison et al. (2019) demonstrated that changes in the gut microbial communities take place in conjunction with their host colonization, adaptation and speciation. They studied the impact of host speciation and divergence on the evolution of its gut bacteria. And their results indicate that the gut microbial communities have shifted by undergoing parallel divergence and speciation to be in synchrony with their stickleback hosts. Thus suggesting the involvement of insect-microbe interactions in driving the evolution of microbial endosymbionts.

While it is true that bacterial composition and structure inside an insect gut is primarily determined by the action of various evolutionary forces acting upon the residing endosymbiotic bacteria and its host, the fact that bacterial populations within an insect body live as a community where they have to share limited resources, cannot be overlooked. Under such a scenario, where the resources are limited, the constant battle for resources amongst the co-residing microbes is bound to occur and as a consequence, inter- and intra-specific competition is inevitable. Though we could not find any direct evidence of such phenomenon occurring inside an insect gut, there are indications from studies on the human-microbe symbiotic association and phytopathogenic bacteria vectored by insects corroborating the above conclusion. Several studies on the human microbiome reveal temporary shifts in the microbiome composition depending upon the dietary intake (Leeming et al., 2019). Recently, it has also been reported that *Gardnerella* subgroups (component of human vaginal microbiome) compete with each other and that in turn affects their population dynamics (Khan et al., 2019). Studies by Jones et al. (2019) indicate that the host plant directly influences the composition of gut microbiota in *Helicoverpa zea*. They have shown that the bacterial communities differ between populations of *H. zea* feeding on different host plants distantly located at different feeding sites. Additionally, a study conducted on the phytopathogenic symbionts that are transmitted by leafhoppers also supports this hypothesis. Rashidi et al. (2014) have shown that though two *Phytoplasmas* are acquired by the leafhopper, *Euscelidius variegates*, during the feeding process, i.e., *Candidatus* Phytoplasma vitis, that causes Flavescence dorée (FDP), and *Candidatus* Phytoplasma asteris, which is the causal agent of Chrysanthemum yellows (CYP), only CYP was efficiently transmitted by the doubly infected leafhoppers. Additionally, it was shown that *P. vitis* was outcompeted by *P. asteris* and hence, was seldom detected in the salivary glands. They conclude that the competition between the two *Phytoplasmas* affected salivary gland colonization by *P. vitis* and during the course of their association with the leafhoppers; *P. asteris* had acquired the greater ability to colonize the insect body and thus ensuring its transmission. Based on these studies, it is plausible to state that microbes compete with each other for survival even within an insect body. And as nutrient accessibility is a major limiting factor, therefore, it is reasonable to believe that the predominance of microbes within an insect body could also be determined by the availability of nutrients and their rate of consumption. Additionally, this could also account for microbiome fluctuations observed in insects when they feed on resistant and susceptible plants or on recalcitrant food sources. Here, it is speculated that under certain conditions, microbes can co-exist (e.g., nutrient-rich conditions) while under other conditions (e.g., nutrient-poor conditions) the specific taxa are outcompeted due to acute nutritional limitations. This would imply that the “resource ratio” competition model, which was first proposed by Tilman (1977) based on the work on plankton algae and later on was reported to hold true for various bacterial communities thriving together, is likely to hold true for insect gut endosymbionts as well. However, additional experimental verification would be required to prove this hypothesis.

## Implications of Genome Size Reduction for the Endosymbiotic Bacteria

Bacterial population that is continuously experiencing genome degradation (either due to the selection pressure or as a consequence of genetic drift) cannot escape extinction. Ultimately, a critical stage of genome erosion is achieved; wherein obligate endosymbionts start suffering from ‘genome reduction syndrome’ (Latorre and Manzano-Marín, 2017) that symbolizes their evolutionary “dead-end.” Extreme gene losses lead to complete dependency of these bacteria on one another and/or their host, as they are incapable of surviving on their own (Husnik and Keeling, 2019). While reducing the metabolic versatility of these endosymbionts, on a long-term evolutionary scale the bacteria with reduced genome have less flexibility and thus lower chances of survival (as compared to their wild-type forms), especially during a sudden environmental change. Although gene loss increases the dependency of the symbiont on the host while reducing the cost associated with symbiosis, excessive genome decay leads to a point where the bacteria is unable to maintain “healthy” association with its host, i.e., it becomes incapable of fulfilling host requirement (Latorre and Manzano-Marín, 2017). Under such a scenario, the bacterial population either suffers a collapse or is marked for replacement. Vogel and Moran (2013) have already shown the replacement of *Buchnera aphidicola* by yeast-like endosymbionts in *Cerataphis brasiliensis*. However, recently the second case of loss of this ancient endosymbiont *Buchnera* from the members of aphid genus *Geopemphigus* has been documented. Here, *Buchnera* was found replaced by another symbiont from the bacterial phylum Bacteroidetes (Chong and Moran, 2018).

So how do symbionts avoid such an evolutionary scenario? One possible strategy to escape extinction is to replace the inefficient bacterium with its free-living counterpart every now and then. This is possible for facultative symbionts and the ones that are environmentally acquired or horizontally transmitted. But strict vertical transmission of some bacterial species becomes a major obstacle for many obligate symbionts. Until recently, it was a puzzle as to how these obligate symbionts managed to survive for long periods in an insect gut. However, it has been now shown that these ancient symbionts establish a di-symbiotic relationship with newly acquired bacterial species. Manzano-Marín et al. (2020) have reported that *Erwinia*, (which is a newly acquired symbiont of aphids) complements *Buchnera* (an ancient symbiont) by serial horizontal transfer of several vitamin biosynthesis genes and thus, compensating for the massive gene loss undergone by *Buchnera* during the long period of its association with its insect host. Similarly, a horizontal gene transfer event was observed between *Cardinium* and its donor organisms, *Wolbachia* and *Rickettsia*, which counterbalance the significant gene loss undergone by *Cardinium* to adapt to the gut environment of its host (Zeng et al., 2018). In addition, recently it has been demonstrated that genome reduction in bacterial species is usually preceded by the acquisition of genes, essential for host survival, from other co-residing microbes via horizontal gene transfer. This is evident from the study conducted by Waterworth et al. (2020) where it has been shown that *Burkholderia gladioli*, present in the beetle, *Lagria villosa*, has undergone extensive genome reduction over time. However, to sustain the symbiotic relationship and avoid extinction, it has acquired the lagriamide *lga* biosynthetic gene cluster, required to augment the metabolic pathway of the host, from other associated symbionts. Furthermore, in some cases, the obligate symbionts are highly reliant on the facultative symbionts for their survival, especially, under extreme conditions. Recently, it has been shown that aphid populations upon exposure to high temperature have reduced lifetime, fecundity and population densities of both obligate and facultative symbionts. However, this reduction is significantly less in aphids that are infected with either of the two facultative symbionts *Regiella insecticola* or *Fukatsuia symbiotica*. Moreover, it was observed that the reduced population density of the obligate symbiont, *Buchnera*, as a result of heat shock, could be successfully recovered in aphids infected with *Regiella* or *Fukatsuia*, but not in uninfected insects (Heyworth et al., 2020). Thus implying that sensitivity of *Buchnera* to heat shock, as a consequence of extreme gene loss, is compensated by the co-residing facultative symbionts.

### Could These Changes Lead to Speciation of Bacterial Species Within Insect Gut?

Considering that genome re-arrangements and major genome deletions are known to occur in the microbial genomes within an insect, an obvious question that arises is do new species of microorganism originate within insects? Recent studies have hinted toward the incidence of sympatric speciation of bacterial species occurring within an insect gut. For instance, *Candidatus Hodgkinia cicadicola* has a highly reduced genome and is reported to have split into two interdependent bacterial species in some species of cicadas. However, it is interesting to note that in some cicadas the ancestral type is found to co-exist with its newly evolved form (Van Leuven et al., 2014). As discussed above, the endosymbiotic bacteria undergo massive changes in its genome and experiences high evolutionary pressures within an insect gut, and therefore, it is plausible to believe that these changes are manifested in the form of evolution of new species, i.e., leads to speciation.

In summary, although possessing a dynamic genome facilitates bacterial adaptations to insect gut, it also has certain disadvantages. Though recent studies have unraveled some of the mechanisms evolved by these endosymbiotic bacteria to cope with the repercussions of having an unstable genome, there likely exist several other mechanisms that are yet to be discovered.

## Consequences of Insect–Microbiome Interactions on Insect Hosts

Insect populations are exposed to various types of environmental fluctuations and stresses periodically. And the only way for any organism to survive the extreme conditions is ‘adaptation.’ Though insects are capable of accommodating variations in its genome brought about by changing environmental conditions, these variations could sometimes be deleterious. Under such eventualities, insects can utilize its microbiome as an alternative for ensuring its adaptation, without compromising or putting its survival at stake. Also, it has been observed that the insect’s microbiome is highly dynamic in terms of its structure, function and composition as it experiences high evolutionary pressures within an insect gut. Though there are cases where the primary symbionts, despite possessing a highly reduced genome, are extremely stable in terms of their gene content, still there is always enough scope for rapid sequence evolution between closely related bacterial species. And with the knowledge that changes in microbiome dramatically influence the host physiology, it is reasonable to believe that insects could exploit this genetic variation, present in its symbiotic species, for its own benefit. A direct evidence of such an occurrence comes from the study carried out on polymorphic *Buchnera* populations present in its insect host, *A. pisum.* The *Buchnera* populations displayed polymorphism in the promoter of a heat shock gene, *ibpA*, which affects the thermotolerance of its insect host (Dunbar et al., 2007). Therefore, it appears that the evolutionary changes in the endosymbiotic bacteria have profound implications on host biology. Infection of *Rickettsiella viridis* in the pea aphid, *A. pisum*, is known to remarkably alter the host phenotype. Aphid populations have red and green colored genetic morphs and it is reported that upon infection with *R. viridis*, red aphids become green due to increased production of green polycyclic quinone pigments (Nikoh et al., 2018). This suggests that gut microbiome can drastically influence the phenotype of their insect host.

Interestingly, endosymbionts also modulate the gene expression of their insect hosts for ensuring their survival and persistence within an insect body. *Candidatus* Liberibacter asiaticus alters the energy metabolism of its psyllid vector, *Diaphornia. citri*, in order to secure its own needs. Genome analysis of *L. asiaticus* revealed the presence of an ATP translocase, which is involved in the uptake of ATP and other nucleotides from the medium for its growth and multiplication. To meet its energy requirements, *D. citri* produces ATP and other energetic nucleotides; however, their utilization by the insect is competitively inhibited by *L. asiaticus* (Killiny et al., 2017). This suggests that the symbiotic bacteria likely influence the biochemical processes within their insect hosts.

In fact, several changes in the insect genome have also been reported that are crucial to insect-microbe symbiotic relationship. Usually, to combat microbial infections, insects have evolved the Toll-like receptor (TLR) and Immune Deficiency (IMD)-like pathways that are responsible for the immune response that functions through the production of antimicrobial peptides (AMPs). For instance, in weevils, the IMD-like pathways are usually involved in secluding the endosymbionts within the bacteriocytes and mediating the systemic and local immune responses to exogenous challenges faced by insects as reported by Maire et al. (2018). Likewise, in the red flour beetle, *Tribolium castaneum*, the IMD pathway was proposed to confer resistance against the Gram-negative and Gram-positive pathogens *Enterobacter cloacae* and *Bacillus subtilis*, respectively (Yokoi et al., 2012); IMD homolog (*Tm*IMD) cloned and functionally characterized from the mealworm beetle, *Tenebrio molitor*, is involved in the expression of nine *AMPs*, which confer resistance against Gram-negative bacteria (Jo et al., 2019). However, in several cases of insect–microbe symbiosis, it is shown that the IMD pathway has been disrupted in insects, and these disruptions likely ensure the survival of its bacterial partners. For instance, the non-functional IMD signaling pathway and absence of several antimicrobial peptides in aphid has probably facilitated the *Buchnera aphidicola*-aphid symbiosis (Gerardo et al., 2010) which originated ∼200 million years ago (Baumann, 2005). Dependence of insects on their beneficial endosymbionts is believed to act as a selective force, which has led to reduction in their immune capabilities. Similarly, *Rhodnius prolixus* has lost several steps critical in the IMD pathway rendering it inactive (Salcedo-Porras et al., 2019); the bedbug, *Cimex lectularius* has a non-functional IMD pathway, an adaptation to prevent elimination of beneficial symbiotic gut microbes (Benoit et al., 2016). This implies that insect hosts too have undergone biochemical and genetic changes to accommodate these beneficial microbes and thereby indicating co-evolution of insect host with its bacterial partner.

In recent years, researchers have also proposed a role for epigenetics in promoting microbial persistence in insects. It is reported that the alternation of DNA methylation patterns by microbes attenuates immune responses in insects and thereby, ensuring the survival of bacterial symbionts (Kim et al., 2016). Additionally, bacterial symbionts with highly reduced genomes have evolved various small RNAs that help them modulate the expression of essential symbiotic genes and regulate core housekeeping processes in their insect hosts (Hansen and Degnan, 2014).

In addition to the biochemical and genetic changes, several behavioral changes in insects could also be attributed to their microbiome (Lewis and Lizé, 2015). In *Drosophila*, it has been demonstrated that gut microbes play a crucial role in determining its behavior and development, as they are involved in the identification of suitable feeding and egg-laying locations. Furthermore, the results of the oviposition assays showed that while exposing *Drosophila* to *Saccharomyces cerevisiae, Lactobacillus plantarum*, and *Acetobacter malorum* promoted its development, exposure to only *S. cerevisiae* and *A. malorum* resulted in the development of larger ovaries and increased egg numbers (Qiao et al., 2019). Further, the microbiome not only influences the host feeding preferences but also determines the insect’s feeding capabilities. For instance, in *Megachile punctatissima* and *M. cribraria*, during egg-laying the females deposit a symbiont-containing capsule that is ingested by the offspring upon emergence. They have evolved this mechanism as a means to exchange bacterial species amongst them. *M. punctatissima* normally feeds on pea while *M. cribraria* is unable to do so. However, when there is an exchange of bacterial species the inability of *M. cribraria* to feed on pea is reversed (Hosokawa et al., 2007). Thus, evidence points toward the role of microbiome in widening host’s niche by allowing its survival on a particular food resource.

Endosymbionts are also known to determine the viable temperature ranges, modulate desiccation tolerance and detoxify xenobiotics for their insect hosts (Lemoine et al., 2020). For instance, it was reported that the microbiome infection frequencies determine the geographic distribution of the chestnut weevil, *Curculio sikkimensis*. It was shown that higher titers of *Sodalis*, *Wolbachia* and *Rickettsia* were present in weevils found at the localities of higher temperature; lower numbers of *Wolbachia* and *Rickettsia* were detected in the population found in the regions with higher snowfall; and higher *Curculioniphilus*, *Sodalis*, *Serratia*, *Wolbachia*, and *Rickettsia* infections were characteristically present in weevils feeding on acorns than on chestnuts (Toju and Fukatsu, 2011) and thus indicating the involvement of symbionts in expanding or limiting the insects’ abiotic niches. Based on the above examples, it would be reasonable to state that the microbiome impacts the insect’s ability to colonize and invade varied ecosystems on Earth.

## Indications for Exploiting Microbiome – A Promising Approach Toward Sustainable Pest Control

With the recent advances in science and technology, we have made significant progress in the transformation of agricultural and horticultural industry and thus ensuring self-sufficiency in food production in several parts of the world. However, with rapidly increasing population coupled with rising demand for food, feed, fodder along with a gradual decline in the area under cultivation, have brought out new challenges that are threatening food, nutritional and livelihood security, globally. Though we have made remarkable progress in increasing food production, it is ironic and unacceptable that malnutrition is still widespread in various parts of the world especially in the under-developed and the developing countries. According to the recent World Resources Institute [WRI] (2019) report, food demand is expected to increase anywhere between 60–90% by 2050 due to exponentially increasing human population. Therefore, one of the major global challenges is to be able to meet the rising food requirements of a rapidly growing population. Although crop production is adversely affected by numerous biotic and abiotic factors, agriculture suffers an annual yield loss of ∼20–40% due to insect pests alone (FAO, 2019).

Several pestilent outbreaks of insect pests of agricultural importance can be prevented if such occurrences can be predicted. However, lack of proper forewarning systems and coupled with indiscriminate use of pesticides and excessive use of nitrogenous fertilizers (facets that have become an integral part of the modern agricultural practices), further compounds the problems faced by farmers. To develop an alternative to conventional pesticides, various companies have introduced low dosage molecules in the market but they are neither cost-effective nor easily accessible and moreover, conventional pest-management strategies are proving ineffective. Additionally, invasive pests are one of major problems faced by farmers, globally. Biological control of these ‘alien’ pests is often not possible as the natural enemies that would keep their population size under control are normally left behind in their aboriginal home or at their native place. In spite of the availability of modern agricultural techniques and practices for controlling such pests, which are effective up to a certain extent, they often have many ecological and environmental repercussions. Therefore, devising a pest-management strategy without compromising the sustainability of agro-ecosystems is a major challenge. Researchers have shown that extensive genome degradation makes the obligate symbionts more sensitive to environmental fluctuations than the host itself. The southern green stinkbug, *Nezara viridula*, depends on a specific Gammaproteobacterial symbiont with a highly reduced genome for its normal growth and survival. Severe gene loss has made this symbiont highly sensitive to temperature fluctuations and even small shifts in temperature would kill these symbionts and, eventually, their hosts as well (Kikuchi et al., 2016). Thus, imposing restrictions on the insect in its ability to colonize inhospitable niches. In such cases, limitations imposed by obligate symbionts may help counter the spread of invasive pests and restrict the geographic reach of invertebrate species.

As indicated by their rapidly changing population structure, the insects are evolving at a much faster rate than their host. And, changing climatic and environmental conditions act as a trigger for inducing these changes in insect pests. Based on literature, it would be appropriate to state that throughout its history, microbes have played a very crucial role in insect survival. Furthermore, as gut bacteria experience high evolutionary pressures within an insect body, it seems a likely candidate that facilitates quick adaptations of the insect host to the ever-changing environment. Moreover, owing to the relatively shorter life cycle, the symbiotic bacteria can adapt more readily than the invasive insects to the new environments (Lu et al., 2016). Therefore, merely focusing on plant-insect interaction would be insufficient as insects share an intimate association with its gut microflora that influences the colonizing capabilities of insects. Moreover, understanding the evolutionary trajectory of insects would enable us to determine their population structure and predict their likelihood of invading a particular area.

Indications that the microbiome could be exploited for insect control also comes from various studies conducted on insect that pose serious risks to human health. Insects like wasps, hornets and bees can cause a severe, and sometimes lethal, allergic reaction in humans. Moreover, mosquitoes are known to vector several deadly viruses such as the Zika virus, the Dengue virus, and the West Nile virus. Therefore, significant efforts have been made to control their spread and manipulation of microbiota is emerging as a novel and promising approach to vector control (Scolari et al., 2019). For example, it has been recently shown that wMel strain of *Wolbachia* induces cytoplasmic incompatibility and when introduced into *Ae. aegypti*, it negatively impacts its ability to act as a vector for the Dengue virus (Thomas et al., 2018; Ross et al., 2019). Additional strategies have been developed for identifying and disrupting natural symbionts of mosquitoes such as *A*. *gambiae* or alter them genetically to express anti-pathogen effectors (Wang and Jacobs-Lorena, 2013). Fisher et al. (2017) demonstrated that removal of the vertically transmitted obligate symbionts from insects results in reduced fitness and this reduction is twice as large as that observed with horizontally transmitted symbionts. Moreover, this increases to three times if the symbiont is involved in providing nutritional benefits to the host. Therefore, understanding the nature of insect-microbial symbiosis and targeting the primary symbionts could prove to be an efficient strategy to control the spread of harmful pests.

Some phytopathogenic bacteria, especially those belonging to the family Enterobacteriaceae, were initially insect commensals (i.e., non-harmful associates) but now have evolved into plant pathogens following repeated inoculations into the phloem by their insect hosts during feeding. Therefore, unraveling the interactions established between phytopathogenic bacteria and insect symbionts could also offer a promising tool to impair and therefore, control the transmission of phloem limited plant pathogens in a sustainable and environment-friendly manner (Gonella et al., 2019). Moreover, it has been shown that the tripartite interactions between insects, microbes and plants contribute to the success of various coleopterans such as the Colorado potato beetle (*Leptinotarsa decemlineata*), cereal leaf beetle (*Oulema melanopus*), western corn rootworm (*Diabrotica virgifera virgifera*), red flour beetle (*Tribolium castaneum*), the rice weevil (*Sitophilus oryzae*) and several others (Wielkopolan and Obrêpalska-Stêplowska, 2016). Therefore, understanding and acquiring knowledge regarding the role of insect-associated microbes would be extremely useful in the development of effective control strategies for crop protection against these economically important agricultural pests.

While significant efforts have been made to develop elite plant varieties of crop plants that can tolerate or resist insect attacks, it is well documented that resistance is often not durable. Within a few generations, the insects are able to successfully overcome host defenses and ultimately the plant succumbs to the insects. Despite the progress that we have made in the area of insect–plant interactions, the mechanisms operating in insects, that endow them with the trait of adaptation under stress, are still unclear. Therefore, under the circumstances, it is pertinent to study and unravel and eventually exploit these mechanisms to devise a long-term pest control strategy. As it would be clear from the evidences presented here, the gut microbiome can dramatically influence the physiology, behavior, and genetics of its insect host, and therefore, targeting the microbiome could be counted as an effective approach for developing an integrated, environment-friendly and a sustainable pest-management strategy.

## Author Contributions

AG and SN contributed to the conception, wrote the manuscript, participated in the writing and analysis, and read and approved the final manuscript. Both authors contributed to the article and approved the submitted version.

## Conflict of Interest

The authors declare that the research was conducted in the absence of any commercial or financial relationships that could be construed as a potential conflict of interest.
