# Supplementary material for: Dynamics of Insect–Microbiome Interaction Influence Host and Microbial Symbiont
Source: Front Microbiol. 2020 Jun 26;11:1357. doi: 10.3389/fmicb.2020.01357 (PMC7333248; doi:10.3389/fmicb.2020.01357)
Supplement: Supplementary file 1 [file Table_1.DOCX]

Supplementary Table T1: Genome sizes (in kb) of members of the insect microbiome when found in obligate or facultative relationship with its host or when in a free-living state.

| **Genus** | **Genome size (Kb)** | | | **Insect Host** | **Reference** |
| --- | --- | --- | --- | --- | --- |
|  | **Obligate** | **Facultative** | **Free-living** |  |  |
| *Ishikawaella*  *Blochmannia*  *Wigglesworthia*  *Baumannia*  *Arsenophonus*  *Blattabacterium*  *Moranella*  *Profftella*  *Buchnera*  *Portiera*  *Uzinura*  *Sulcia*  *Carsonella*  *Hodgkinia*  *Tremblaya*  *Hamiltonella*  *Regiella*  *Serratia*  *Endolissoclinum*  *Sodalis*  *Erwinia*  *Ruthia*  *Wolbachia*  *Nardonella*  *Rickettsia* | 600-750  700-792  700  (50-80 mya)  633-700  570  638  538  460  416-644  357-360  263  191-277  173  <150^a^  (260 mya)  138-143  -  -  650-1760  (>100 mya)  1480  2000  1100  1160  900-1100  232  1100 | -  -  -  -  3500  -  -  -  -  -  -  -  -  -  -  1840  2100-2400  2790-3580  -  4500  -  -  1250-1500  -  2100 | 1200  (Ancestral genome)  -  -  -  -  -  -  -  -  -  1100^b^  -  -  -  5110-5450  >6000  4550  3830-5070  2200  -  -  - | Stinkbugs  Carpenter ants  Tsetse flies  Sharpshooters  Various insects  Termites  *Tremblaya* (within bugs)  Psyllids  Aphids  Whiteflies  Armoured scales  Leafhoppers  Psyllids  Cicadas  Mealybugs  Aphids, Psyllids, Whiteflies  Aphids  Pea aphids  Asian citrus psyllids  Tsetse flies  Aphids  Clams  Various insects  Weevils, Beetles  Various insects | Nikoh et al., 2011  Zeintz et al., 2006  Akman et al., 2002  Bennett et al., 2016a  Nováková et al., 2016  Patiño-Navarrete et al., 2013  Husnik & Mc Cutcheon, 2016  Nakabachi et al., 2013  Chong & Moran, 2018  Santos-Garcia et al., 2015  Bennett et al., 2014  Sabree et al., 2013  Bennett et al., 2016b  Katsir et al., 2018  ^a^Campbell et al., 2015; ^b^Van-Leuven et al., 2014  López-Madrigal et al., 2011  Rao et al., 2012  Hansen et al., 2012  Burke & Moran, 2011  Nikoh et al., 2019  Kwan et al., 2012  Akman et al., 2001  Manzano-Marı́n et al., 2020  Roeselers et al., 2010  Miller, 2013  Kuriwada et al., 2010  Bishop-Lilly et al., 2013 |

mya: million years ago

- : Data not available
